# Regional trends in extended-spectrum beta-lactamase-producing enterobacteriaceae (ESBLE), E. coli SBLE and K. pneumonia SBLE between 2009 and 2013

**DOI:** 10.1186/2047-2994-4-S1-O44

**Published:** 2015-06-16

**Authors:** I Arnaud, V Jarlier, P Astagneau

**Affiliations:** 1 French healthcare associated infection control Network (CClin-Arlin), France

## Introduction

Extended-spectrum beta-lactamase-producing Enterobacteriacae (ESBLE) (especially *E. coli* and *K. pneumonia* (Kp)) infections have dramatically increased for the last 10 years in many countries. In France, despite control efforts with specific guidelines and promotion campaign towards hospitals, the upward trends remains ongoing.

## Objectives

The aim of this work is to determine whether regional variations could be identified according to ESBLE species.

## Methods

A cohort of 577 Health care facilities (HCF) from 2009 to 2013 is issued from the national monitoring network of multidrug resistant bacteria in hospital (BMR-RAISIN) implemented since 2002. HCF participated within a 3 month-period on a voluntary basis. Strains were isolated from sample issued for diagnostic purposes (a single strain of the same species per patient). Incidence of ESBLE stratified by region was calculated per 1,000 patient-days (PD) from 2009 to 2013. Poisson regression was used to estimate temporal trends.

## Results

From 2009 to 2013, the incidence of E. coli increased from 0.19 to 0.32 per 1,000 PD. The same upward trends were observed for Kp (0.05 to 0.13 per 1,000 PD) and *E. cloacae* (0.04 to 0.06 per 1,000 PD). Conversely, the incidence of other ESBLE species including *Enterobacter aerogenes* tended to decrease. ESBLE bacteraemia incidence increased from 0.03 to 0.05 per 1,000 PD (425 to 704 bacteraemia), representing a 77% increase (p<0.001). ESBLE incidence increased nationwide but varied according to regions (median P value of Poisson regression test: 10-3, range [0.37 -10-3]). The highest were observed in Eastern regions (including Paris area, +233%) whereas the lowest in Western regions (+28%). In 2013, incidence was greater than 0.35 per 1,000 PD in all regions except Western regions. A similar distribution was found for *E. coli*, with lowest incidence in West and highest in Paris area (>0,4). The North and South West region have an highest incidence for Kp.

## Conclusion

Large geographical variation of ESBLE trends were observed, emphasize the need to reinforce control measures focusing on high incidence areas.

## Disclosure of interest

None declared.

